# Novel clip applicator for minimally invasive surgery

**DOI:** 10.1007/s00464-019-06860-5

**Published:** 2019-06-21

**Authors:** Simon Erridge, Christopher J. Payne, Mikael Sodergren

**Affiliations:** 1 Department of Surgery & Cancer, Imperial College London, London, UK; 2 John A. Paulson School of Engineering and Applied Sciences, Harvard University, Cambridge, USA; 3 Academic Surgical Unit, Division of Surgery, Department of Surgery & Cancer, Imperial College London, 10th Floor QEQM, St Mary’s Hospital, South Wharf Road, London, W2 1NY UK

**Keywords:** Surgery, Endoscopy, Technology, Laparoscopy, Surgical devices

## Abstract

**Background:**

Ligation clips are used ubiquitously throughout minimally invasive surgery for apposition of tissues. Their size limits their application beyond ligation of small tubular structures. A novel clip and clip applicator that allows for broad-area clamping and rotation has been developed by our team. The primary aim of this study is to provide preliminary data assessing tensile strength of the clip across apposed segments of bowel.

**Methods:**

A comparative study evaluating the maximum load (N) held across two apposed tissues by (a) our novel broad-area clip and (b) a conventional commercial clip was performed. Two sections of porcine bowel were clamped together and the maximum load (N) was measured using a tensile strength material testing machine. A preliminary experiment comparing staple line leak pressures in a porcine model ± clip enforcement of staple line was also conducted. *p* < 0.05 determined statistical significance.

**Results:**

Twenty-four samples (intervention = 15; control = 9) of porcine bowel annealed by surgical clips were tested. The mean maximum force withheld by the bowel and staples was greater for our novel clip design (2.043 ± 0.831 N) than the control clip (1.080 ± 0.466 N, *p* = 0.004). Ten staple line (intervention = 5; control = 5) pressures of porcine bowel were measured. There was no statistically significant difference between the leak pressures with clip reinforcement (84.8 mmHg; range 71.8–109.8 mmHg), or without (54.1 mmHg; range 26.3–98.9 mmHg).

**Conclusion:**

These preliminary results suggest that our novel clip is able to withstand higher tensile force across tissues compared to a leading commercial clip. A small preliminary trial of effect on leak pressures demonstrated no statistical significance; however, increasing reliability of staple line deformation may be a clinically important finding. Whilst further iteration of product design and clinical testing is required, this product may occupy an important clinical niche through staple line reinforcement, enterotomy closure and other applications.

The modern surgical ligation clip was borne out of an invention by Ernest C Wood in 1968 [1]. His patent for a ‘haemostatic clip’ has since been cited in 124 separate patents for surgical clips and clip applicators of various descriptions. This has helped revolutionise both open and minimally invasive surgery. Ligation and haemostatic clips work by approximating tissues to one another. This has notably provided most benefit when applied to small tubular structures such as blood vessels to provide haemostasis or to occlude anatomical ducts. In laparoscopic surgery, where manual dexterity can be limited by technical restraints, a device that can provide quick and easy haemostasis is of great benefit to the operating surgeon [2].

Conventional laparoscopic clip application systems have noteworthy limitations despite their ubiquitous use. Firstly, most commercially available clips only have a nominal width of approximately 1 mm [3]. This makes them ideal for ligating small tubular structures, however, their size limits any additional functionality. There is a growing desire within the surgical community to find novel applications and adaptions to existing surgical devices to improve clinical practice. A notable example can be found in bariatric surgery where mechanical stapling devices are the mainstay of sleeve gastrectomy operations. Perioperative leak and haemorrhage at the gastric staple line are significant causes of morbidity and mortality, despite the sleeve gastrectomy’s otherwise favourable safety profile [4–6]. Staple line reinforcement (SLR) with buttress material was developed to reduce the incidence of these complications. However, there is conflicting evidence to support its routine use in preventing staple line leaks and haemorrhage, with some studies even suggesting worse outcomes with SLR [4–7]. SLR also increases the average intraoperative costs associated with sleeve gastrectomy by €746 per procedure [8]. Due to the size of currently available surgical clips their use in preventing or treating staple line complications is limited as they can only treat small, discrete areas (Fig. 1). This is the inspiration for the development of our novel surgical clip, which has a width of 15 mm (range 8–24 mm) allowing for approximation of larger areas of tissue. This is combined with our unique laparoscopic clip applicator that rotates the clip through 90° in the horizontal axis aiding precise application.

**Fig. 1:**
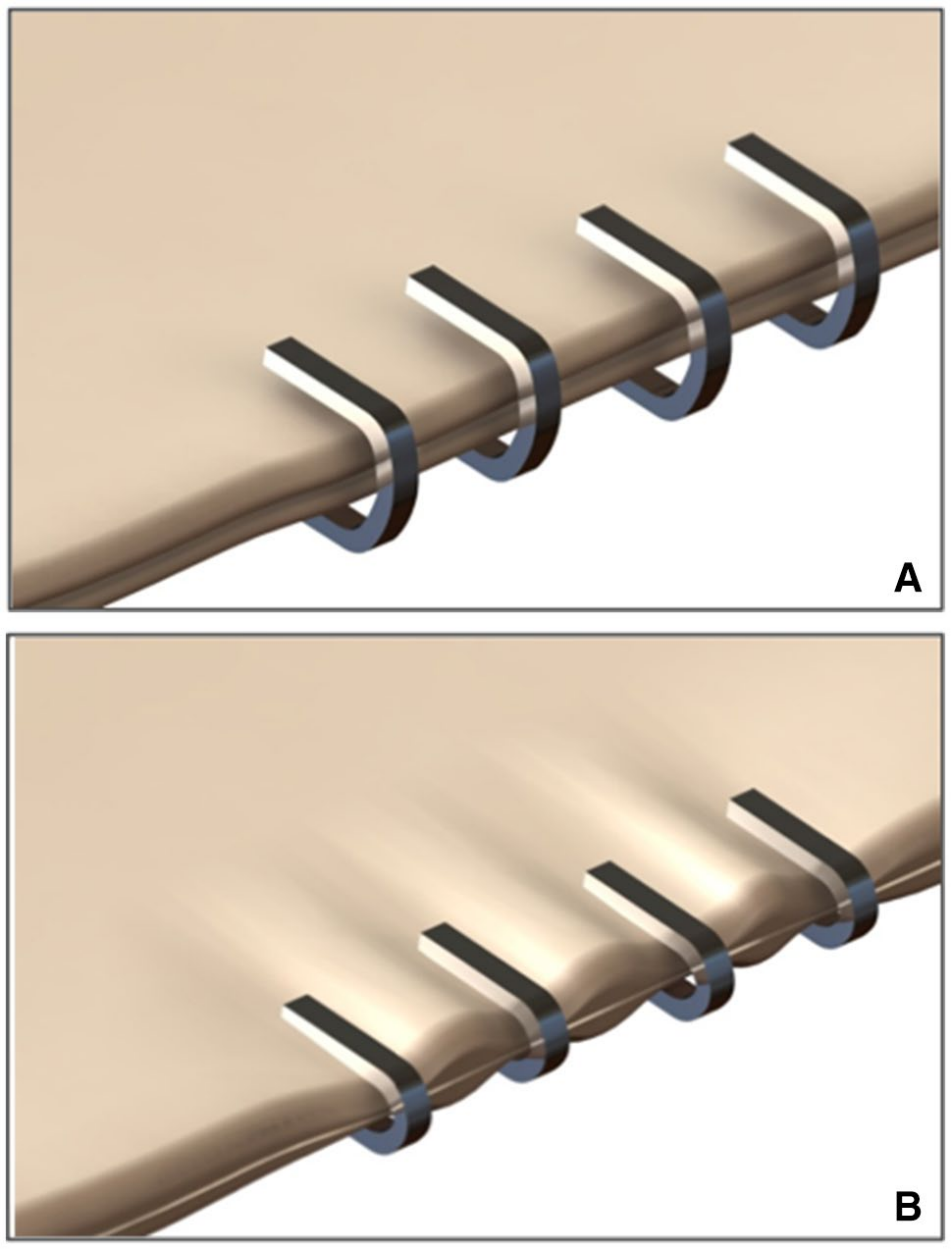
Traditional clips used for tissue approximation have notable limitations when used across a large area (left: before and right: after, clipping tissue)

Despite the initial inspiration of our novel design being for abutment or haemostasis of sleeve gastrectomy staple lines, it has become clear that it could have applications far exceeding this. Enterotomy closure is a technically challenging laparoscopic skill, especially to achieve symmetrical tissue approximation [9]. Haemostatic clips have been used off-license for enterotomy closure in flexible endoluminal surgery [10], but their dimensions have prevented adoption in laparoscopic surgery. Our clip design could overcome this not only the elective setting but also in the treatment of peptic ulcer perforation and fistulas. Wide clips coupled with an articulating applicator also have potential to further advance flexible endoscopic surgery where limits in surgical device translation have limited its widespread implementation [10]. This invention therefore represents a paradigm shift and completely new concept in surgical tissue approximation.

Surgical innovation has been scrutinised for its previous disregard for transparent academic review before widespread adoption. The IDEAL framework sets out that the first step in assessment of surgical devices is a staged multidisciplinary pre-clinical programme of research [11]. This study presents the initial pre-clinical testing of the novel clip and its applicator. The primary aim of this study is to compare the tensile strength of our elongated clip as applied to porcine bowel against a commercially available clip. The secondary aim is to assess if additional reinforcement of the staple line with an elongated clip can improve the leak pressure threshold at the staple line. This will form the basis for aiding translation from bench to the operating room.

## Methods

### Device development

Our team at Imperial College London, led by Mikael Sodergren, developed the novel clip and its applicator. The invention has been in development from conception in 2012, with a first prototype created in 2016. It is protected by an international patent (WO2018/069690) filed through Imperial Innovations.

Development took place over a number of iterative stages. Initially, a reliable clip manufacturing technique had to be developed using a small-scale batch process. Then a clamping jaw that accommodated rotation and reliably performed clip applications was developed in order to test clip prototypes. The rotatable clamping jaw was subsequently miniaturised and integrated into a sterilisable laparoscopic instrument for pre-clinical trialling.

### Clip design

**Fig. 2:**
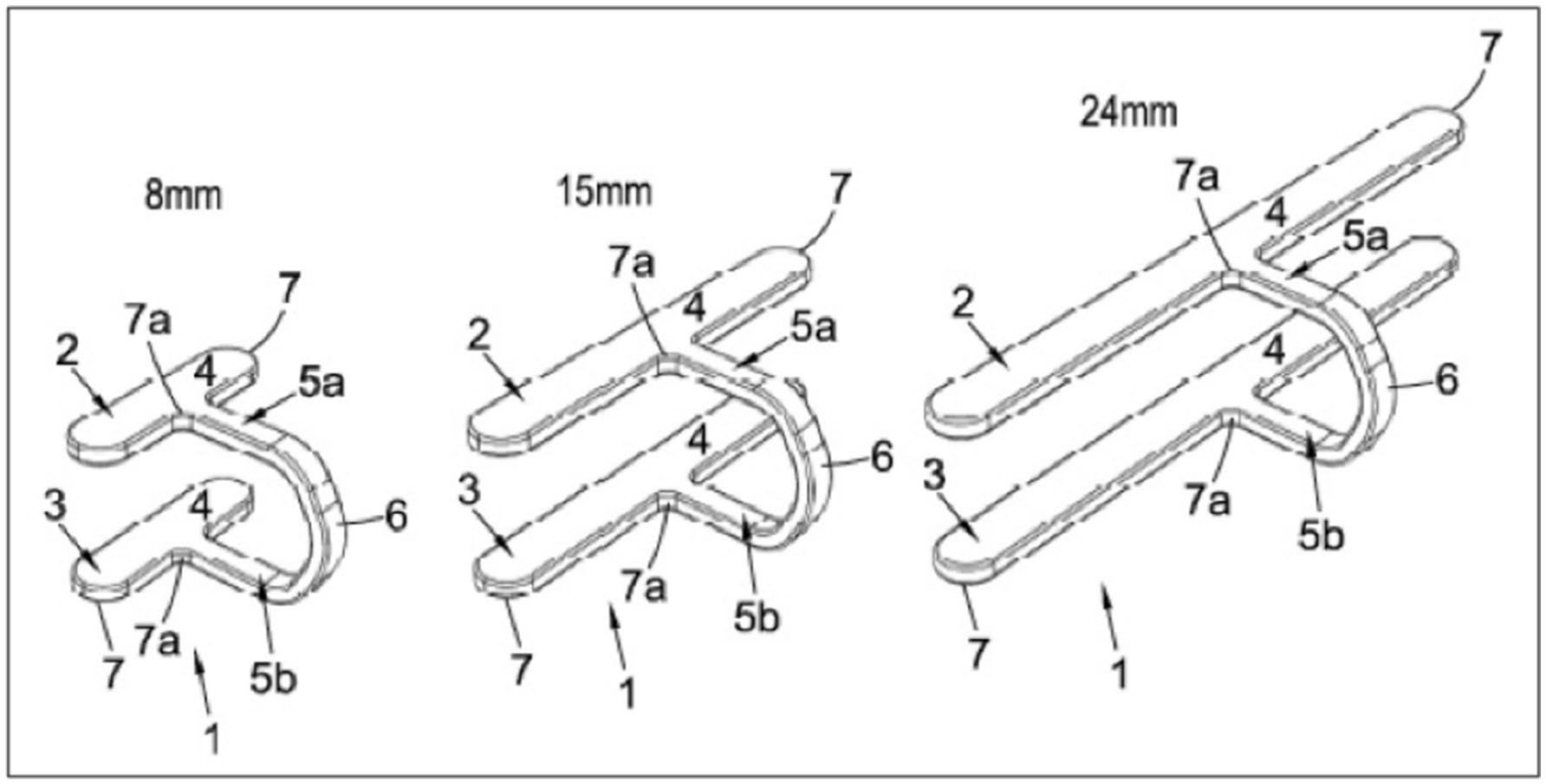
Novel clip design of different size ranges (1)—A superior bar (2) and an inferior bar (3), each of together form an elongated clamping bar (4). In the same plane, for each clamping bar there extends a bracing bar (5a, 5b), which extends perpendicularly to the elongate clamping bar and is typically centrally positioned along its length. The bracing element (6) of the fastener is moulded to join both clamping bars. The edges of the bar (7, 7a) are curved and blunt to reduce risk of perforating tissue

**Fig. 3:**
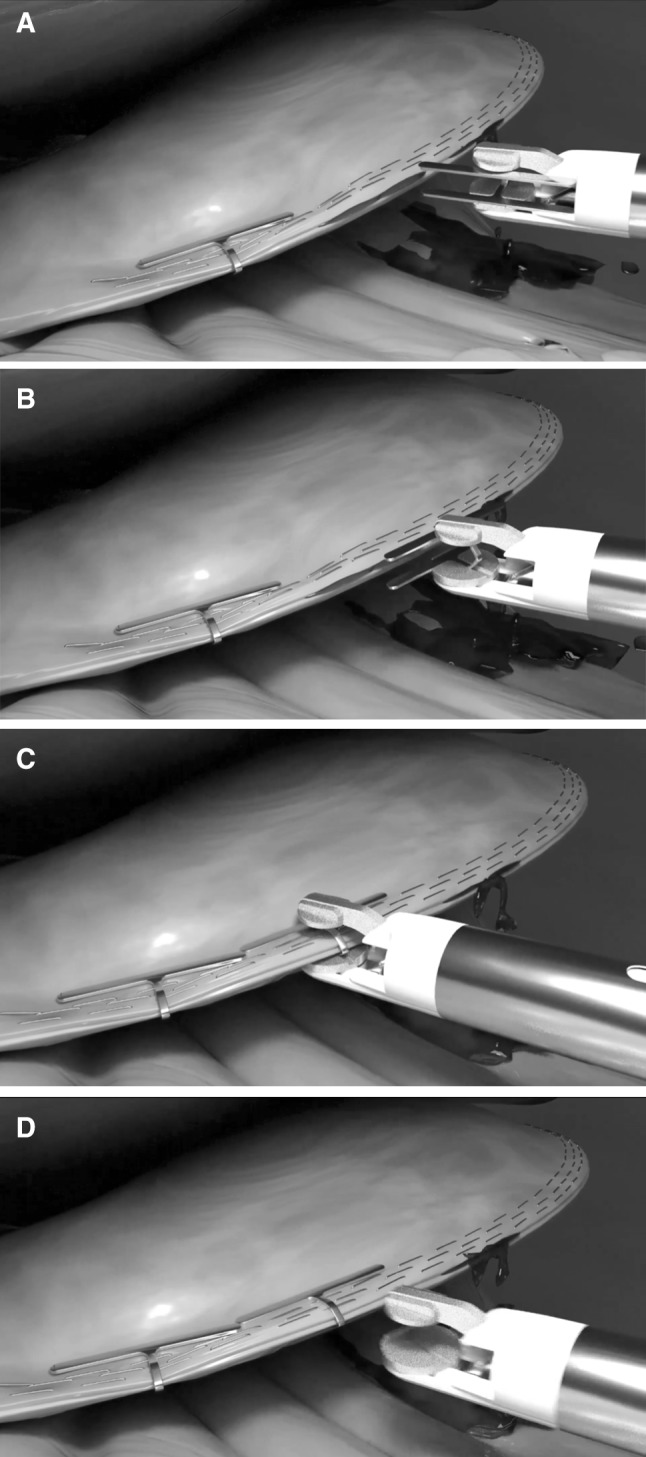
**A–D** Increased width of surgical clip increases potential applications—including staple line reinforcement and enterotomy closure. **A** Applicator inserted with clip bar parallel to the clip applicator axis. **B** Clip is rotated. **C** Tissue is clamped and **D** applicator is retracted

There are a number of novel features pertaining to our clip design. The clip is single-braced with a T profile consisting of two ‘clamping bars’ with a brace to which it is loaded onto a clip applicator (Fig. 2). The clip size measures 10 mm × 2.5 mm × 15 mm (length × depth × width). Using a batch making process, the clip size can also be adapted to a variable range of widths (8–24 mm). These clips provide a similar aperture compared to popular commercial clips (10 mm) such as a standard Ethicon, Inc (Johnson & Johnson, USA) Ligaclip—10 M/L. However, the mean width of those clips is 1.1 mm [3]. Whilst traditional clips provide adequate size to ligate tubular structures, they are limited in compressing significant areas of tissue such as in staple line reinforcement or enterotomy. The increased width of this design would help overcome this (Fig. 3).

The design of most commercial clips aims for a ‘tip-to-tip’ closure. This causes the clip to deform from a ‘V’ into a ‘chevron’ shape during firing. Subsequently, unless the cartridge, applicator and tissue are all perpendicular the clip may deform leading to inadequate tissue approximation. Our proposed design incorporates a reinforcement flange to enhance the rigidity of the clamping bar feature (Fig. 2), as the remainder of the clip will require the ductile properties of standard clips to facilitate application. Ensuring that the clip deforms in a predictable and reliable manner as well as remaining parallel under deformation was paramount when considering this design.

Complementary to this, the clip is designed to help provide rotation to improve tissue approximation. To achieve this, the clip has a ‘dimple feature’ (Fig. 2). This forms a pivot point about which the clip can be rotated in the jaws of the applicator. This feature not only allows for the clip to be retained in the jaws of the applicator but also generate the torque required to rotate the clip.

### Clip applicator design

**Fig. 4:**
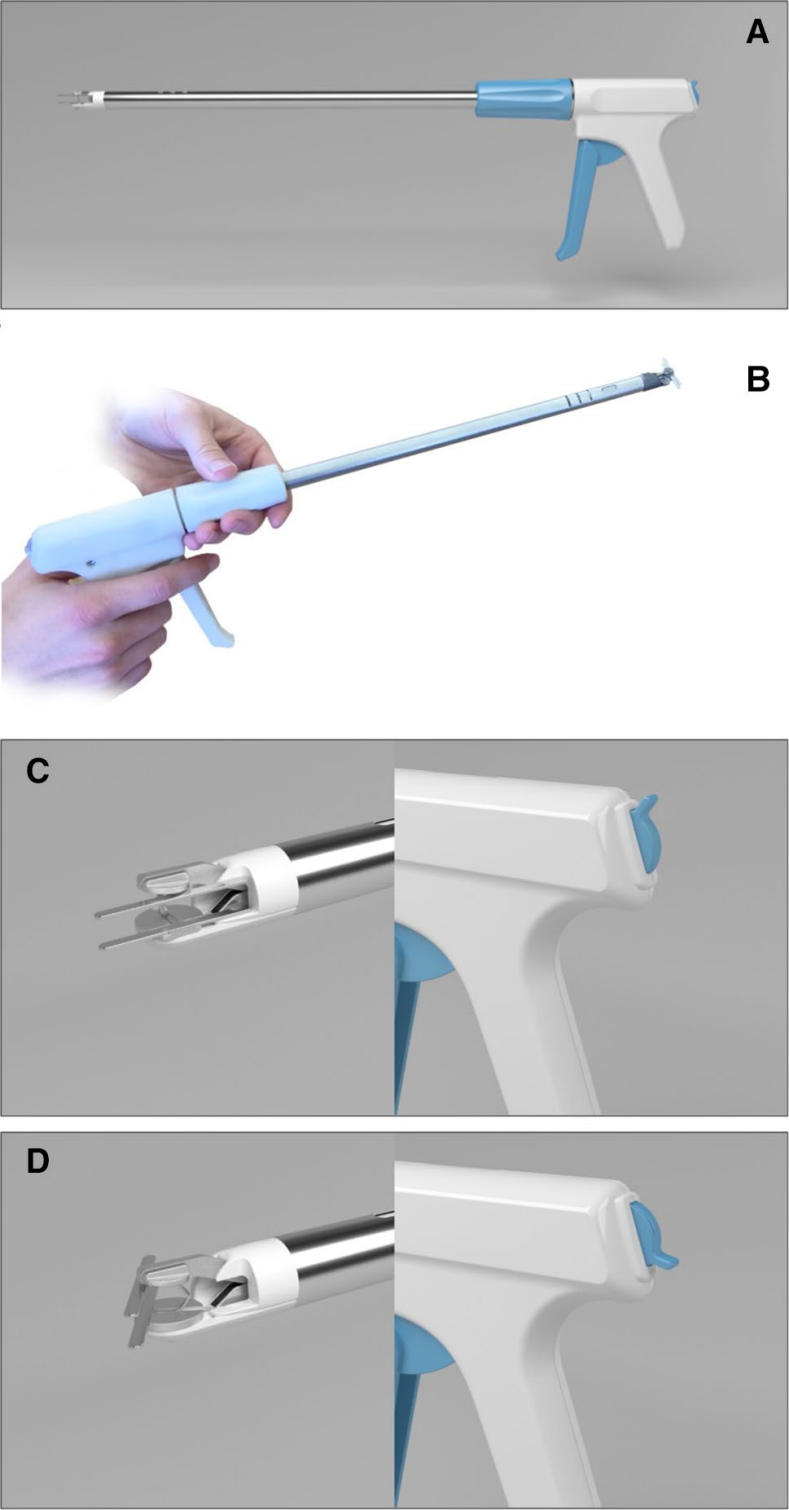
Clip applicator. **A** Full-length computer generated image. **B** Full-length prototype picture. **C** Clip in neutral position for insertion through trocar with lever appropriately positioned. **D** Clip rotated for application to tissue using lever on end of device

Supplementary to the clip itself, the applicator also presents an original innovation of design. Whilst traditional designs allow 360° of rotation of the shaft, our design has the added capability of 0°–110° of rotation of the clip around a pivot (Fig. 3). This is carried out using a tendon-based system that is controlled using a lever located at the back of the handle for ease of use (Fig. 4). The clip is held on spring-loaded jaws to enable precision firing on holding down the trigger. Whilst the applicator has been designed in parallel with clip design, the same design would be capable of applying clips of a variety of sizes.

### Prototype testing

The prototype design had been tested in a box trainer to examine for effects under repeated firing. This demonstrated no depreciable value in quality of clip application and that the clip continued to deform in a predictable and reliable manner. It also demonstrated reliability in clip application between 60° and 120°.

### Experiment 1: Clamping tensile strength

#### Study design

A comparative study was conducted to determine the difference in clamping strength between the elongated clip and a control, a popular commercial clip. The trial was conducted under controlled laboratory conditions at Imperial College London. Institutional review board approval and written consent were not required in this study.

**Fig. 5:**
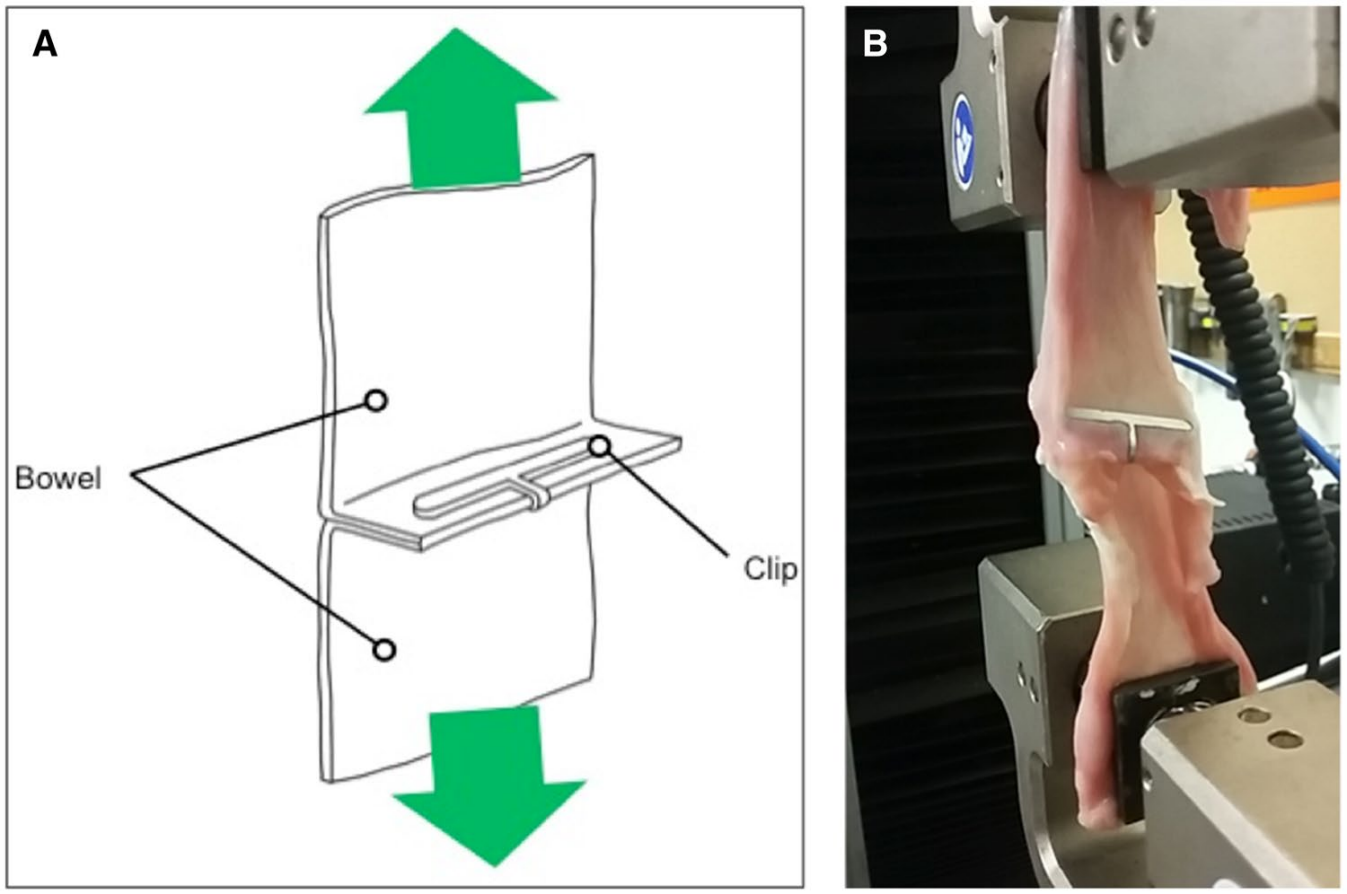
**A** and **B** Two pieces of porcine bowel attached by a surgical clip. Tension is applied to it through a tensile strength material testing machine to measure the peak force it can withstand (Instron™ 5565, Instron, USA)

For the trial, two sections of porcine bowel (approximately 2 mm thickness) were clamped together using surgical clips. The now continuous lengths of bowel were placed inside a tensile strength material testing machine (Instron™ 5565, Instron, USA). The bowel was inserted parallel to the machine to ensure a one-dimensional axial force was loaded through the bowel to prevent any bending forces. The porcine bowel was placed under increasing tension until the clip failed and the bowel separated. At this point, the test would be terminated (Fig. 5).

#### Intervention

The intervention consisted of a single firing of the novel surgical clip (Dimensions 10 mm(length) × 2.5 mm (depth) × 15 mm (width). The control was a single application of a U-profiled ligation clip (Ligaclip 10-M/L, Ethicon, Johnson & Johnson, USA), chosen as it is one of the most popular clips used in the market and is the most commonly used clip in minimally invasive surgery by general surgeons at our affiliated Imperial College Healthcare Trust. A single application was chosen to illustrate equivalence to an existing clip in early pre-clinical assessment.

An ad hoc assessment of 3 consecutive control clips applied across the bowel was conducted and is reported but was not included within our study protocol. This assessment was conducted to gauge the effect on tensile strength by applying U-profile ligation clips along a width equivalent to our clip design.

#### Primary outcome

The primary outcome of the experiment was to determine the maximum force (N) through which the bowel and clips could withstand before failure.

### Statistical analysis

A *t* test was conducted to identify any statistical significance between the mean maximum forces endured by the novel clip design or control clips. Prior to this, a Levene’s test for equality of variances was carried out to determine the variation in each data group. Results are presented as a mean ± standard deviation.

### Experiment 2: Leak pressure threshold

#### Study design

Another study was also performed to assess whether additional reinforcement at the staple line using an elongated clip can improve the leak pressure threshold at the staple line.

The methodology was based on the methods set forward by Mery et al. for assessing staple line reinforcement [12]. Freshly excised porcine bowel segments (20 cm), from pigs euthanized after non-gastrointestinal research, were stapled (Covidien Polysorb GIA 75-0.060 Staplerat, Medtronic, Ireland) at the distal end and attached to a length of tubing at the proximal end. An electronic pressure sensor (TruStability™ NSC Series NSCDANN015PAUNV, Honeywell, USA) was connected into the system to measure inflation pressure.

Bowel specimens were filled with water through the tubing to an initial pressure of 10 mmHg. The specimens were then inflated at an approximate rate of 2 mL/s. Pressure data were acquired by a Powerlab system (Powerlab, AD Instruments, New Zealand) and processed in Labchart (Labchart, AD Instruments, New Zealand). Time of was logged using two separate assessment methods (drop in intraluminal pressure indicating staple line leak and visualisation of staple line leak or burst). The leak pressure (mmHg) was calculated as the intraluminal pressure at the time of staple line leak. In the case that the staple line burst prior to leak this pressure was used instead of leak pressure.

#### Intervention

**Fig. 6:**
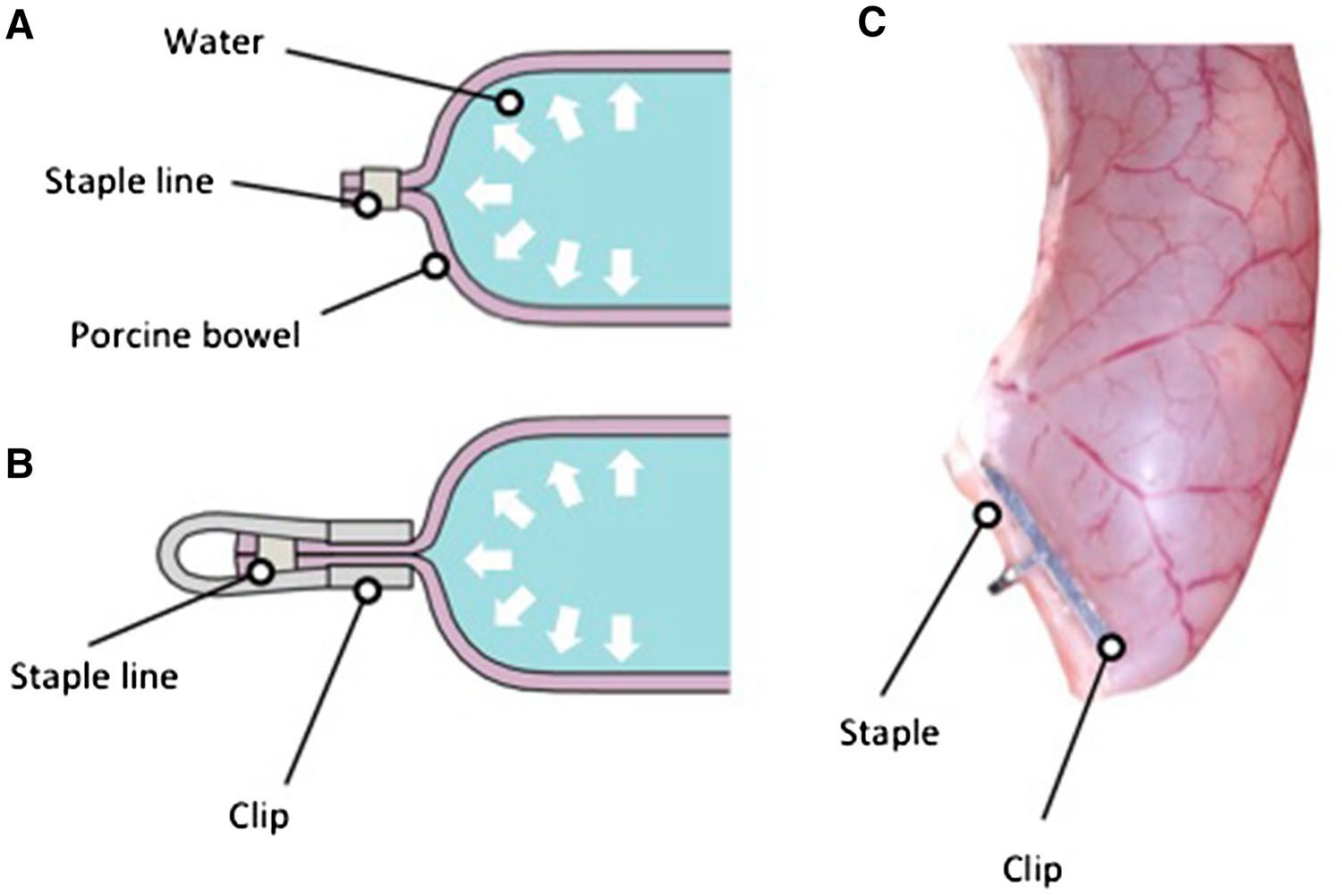
**A–C** Water was used to insufflate a stapled segment of porcine bowel (**A**). Intervention consisted of staple line reinforcement utilising our novel clip design (**B**, **C**). Leak pressure was measured by maximum pressure within the bowel lumen prior to visualised leak at staple line

The intervention group consisted of staple line reinforcement using a single firing of the novel surgical clip (Dimensions 10 mm (length) × 2.5 mm (depth) × 15 mm (width) over the previously formed staple line. The control group consisted of measurement of distally stapled porcine bowel without any staple line reinforcement (Fig. 6).

### Primary outcome

The primary outcome of the experiment was to determine the median pressure a stapled segment of porcine bowel could withstand with and without reinforcement with our clip design.

### Statistical analysis

Leak pressures for each group were calculated as median values and presented with their ranges. Mann–Whitney U test was used to compare the medians of the control and intervention groups. To minimise bias from staple line burst, Kaplan–Meier Curves depicting time to failure were compared for both control and intervention groups. Statistical calculations for both experiments were conducted using SPSS (v.24.0.0.0, IBM, USA). Statistical significance was determined at *p* < 0.050.

## Results

### Experiment 1

Twenty-four samples of porcine bowel connected by surgical clips were subjected to tensile forces (Fig. 5). Fifteen and nine samples were assigned to the intervention and control, respectively. A Levene’s test identified that there was no statistically significant difference in variance between the control and intervention (*F* = 1.264, *p* = 0.273).

**Fig. 7:**
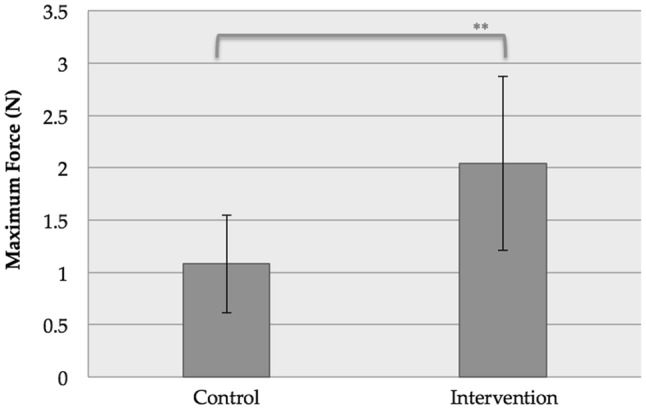
Mean maximum force loaded through either control (Ligaclip 10-M/L, Ethicon, Johnson & Johnson, USA), or intervention (wide clip). **p < 0.01

Analysis of mean maximum force is demonstrated in Fig. 7. The mean maximum force (N) withheld by the bowel and staples was greater for our novel wide clip design (2.043 ± 0.831 N) than the control clip (1.080 ± 0.466 N, *p* = 0.004).

An ad hoc test was run to examine the maximum load through bowel approximated by three separate applications of the control clips. This produced a peak load of 1.497 N. As only one run was conducted it was not included in formal analysis.

### Experiment 2

Leak pressures of ten segments of stapled porcine bowel (control = 5; clip reinforced = 5) were analysed (Fig. 6). Median leak pressures of the control staple line were 54.1 mmHg (26.3–98.9 mmHg). The median leak pressure of the staple line reinforced with an elongated staple clip was 84.8 mmHg (71.8–109.8 mmHg; *p* = 0.117).

## Discussion

The results suggest that our novel clip design can join 2 opposing tissues whilst withstanding higher tensile forces in comparison to a market-leading clip that has undergone numerous iterations and extensive post-marketing feedback. This suggests that the novel design of our clip, particularly its increased width helps improve successful apposition of tissues under tension. The implications of this are that this novel ligation clip may increase the utility of surgical clips in minimally invasive surgery. In particular techniques that require close apposition of tissue such as haemostasis, closure of pancreatic duct following distal pancreatectomy, SLR and enterotomy closure.

The potential applications of our innovative design are widespread. This design still allows the surgeon to provide haemostasis and ligation, articulation of the jaws however allows for improved precision of placement. This is particularly important where inadequate identification of structures and accurate placement of clips can lead to high morbidity, such as in a cholecystectomy [13]. Moreover, as the clip itself is wider and is able to withstand higher peak forces it can help provide reinforcement and haemostasis at staple lines. In particular, reinforcement for staple lines in sleeve gastrectomy is particularly sought after to prevent the morbidity and potential mortality associated with haemorrhage and staple line leak [4, 5, 14]. Whilst SLR has made its way into regular practice, its benefits are unclear with study results demonstrating conflicting data on its protective value against leaks and haemorrhage [5, 6, 15]. The aetiology of these complications is multifactorial but is thought to occur when the pressure of the gastric remnant exceeds the tensile strength of the staple line [16]. Consequently, if our design makes the successful transition to clinical practice, its ability to appose two tissues under higher tension than commonly available commercial clips will likely prove to be beneficial for sleeve gastrectomy patients.

A notable difference beyond the design of the two clips is their material properties. Our surgical clip prototype was made using stainless steel whilst the control clip was made using titanium. Titanium is the most uniformly used metal for intraoperative clips. This is because it is inert, strong and is MRI-compatible [17]. We chose stainless steel clips for initial prototyping and ex vivo testing due to its higher availability to use in a small batch making process, however, the clip could similarly be made using titanium in future iterations.

The second experiment demonstrated no statistically significant difference between leak pressures of stapled porcine bowel with or without reinforcement with our novel clip design. Despite this it did highlight some important properties of staple line reinforcement with our clip. The range of pressures was reduced showing that the staple line deforms in a more predictable manner with staple line reinforcement. Also, all staple lines were able to withstand pressures of 71.8 mmHg and greater with reinforcement, whereas without reinforcement leaks occurred at pressures as low as 26.3 mmHg. This has important clinical importance. A study of intragastric pressures showed that vomiting and retching can produce mean intragastric pressures of 82 and 69 mmHg, respectively [18]. Postoperative nausea and vomiting occur in up to 30% of patients; however, higher incidences of up to 70–80% are observed in high-risk patients undergoing esophago-gastric and bariatric surgery [18–20]. Consequently, staple line reinforcement with a broad surgical clip may yet provide clinical benefit in reducing leak and haemorrhage rates in vivo.

Unfortunately, despite their ubiquitous use in surgical practice, there is a paucity of data detailing surgical clip design, strength and utility. This is a failure of the wider literature, as manufacturing companies receive no benefit from publishing their pre-clinical and clinical results. This limits our ability to further contrast the performance of our clip and its applicator against other devices.

This study is a preliminary investigation of a novel clip and clip applicator in the ex vivo setting and as such is subject to limitations. Our clip was only compared to one of a number of different clips available on the market. Ideally, our clip would be compared against a number of separate clips but resources restricted this. To reduce the limitations of this, we decided to compare our clip against one of the most popular clips used in general surgery procedures. Moreover, the clip was only compared with one application of each clip. Whilst this helps to suggest efficacy per individual clip this is not wholly representative of clinical practice where surgeons may use more than one conventional clip to help oppose two sets of tissue. In addition, our experiment was conducted outside of the clinical setting on porcine tissue. However, whilst this is a limitation, it is an important first step in proving that it may have a valid application before in-human trials. Experiment two was limited by a small sample size. Ad hoc power analysis demonstrated that this was underpowered to detect a significant difference. The study was limited by paucity of materials to conduct further analysis. The results from the first experiment would suggest from an engineering standpoint that if experiment two was well powered a difference would have been detected.

The next step in evaluation of the clip and its applicator involves expansion of the batch making process to enable further pre-clinical trials to be conducted on a larger scale. These shall include a combination of live animal studies assessing necrosis when clamping large bowel areas, as well as surgeon assessment of applicator design and usability. Depending on these studies, the design may continue to undergo further development, iteration and re-examination before beginning in-human trials.

In summary, this study suggests that our novel surgical clip and clip applicator design may in the future occupy an important niche in the surgical device marketplace. These preliminary results suggest that the clip is able to withstand higher tension when used to oppose two pieces of bowel compared to a leading commercial clip. It may also provide a clinically relevant role of improving the reliability of staple line leak pressure. Whilst, further iteration of product design and clinical testing is required this product shows promising potential to be used not only in similar settings to conventional clips but also in staple line reinforcement, enterotomy closure and even management of enteral fistulae and gastric ulcers.
